# Foreign Correspondence

**Published:** 1872-01

**Authors:** A. W. Calhoun

**Affiliations:** Berlin, Prussia


### FOREIGN CORRESPONDENCE.

BERLIN, PRUSSIA, Nov. 13, 1871.

*Messrs. Editors*:—Presuming that you and your readers would have no objection to hearing something of medicine and medical men in this part of the world, I communicate a few points which may be of some interest to you and them, and perhaps, also, some who glance over this, and who are contemplating a visit to the European schools may be in the same quandary as myself, before leaving America, viz: as to selecting the best point for studying general medicine and the different specialties. And if so, I hope in as few words as possible, to give them a few ideas by which they may be better able to guide their movements.

For a number of years Berlin has taken a prominent stand among the European schools, and the results of the recent war has not only had no depreciating effect upon the German Institutions of different kinds, but has acted upon them as upon the Empire itself—made them conspicuous over all others.

The different medical schools are advertised to begin on the 15th of October, but nothing is really done in the way of Lectures or Clinics, till about the 1st of November. They continue regularly to the middle of March, when a recess of six weeks is given, after which they again progress till the 1st of August, when the schools are closed for the three following months.

Students begin to arrive towards the middle of October, and up to this time they have come in such numbers that it is with difficulty that suitable londging can be obtained. In fact, Berlin is one vast school-house, and the pupils hail from almost every quarter of the globe, and are of every color peculiar to man. Aside from the Germans themselves, the Americans exceed all others in numbers, and are applying themselves to the study of every imaginable branch of the profession.

The two main Hospitals are the "Charite," containing 1,800 beds, and "The Royal Surgical Clinic," with 200 beds. Numerous other institutions of similar character are scattered throughout the city, but the above two are the principal ones, and to which all students and visitors attend. The latter is under the exclusive control of Professor Largenbeck, the former has its different departments, managed by different Professors. Military rule, as it does in all things else in Germany, extends also its strong arms through and around these "Homes for the Sick," and as a consequence it is a somewhat difficult task to procure permission to pass through and examine the departments, even in company with the Professors themselves, and as a result of this partial prohibition, one is deprived of one of the most important parts of his study, viz: "Bed-side examinations."

The Clinics are in every respect first-class, and so sought after that the amphitheaters are unable to hold the applicants.

I have made frequent inquiry of the schools of Paris and Vienna, of those recently coming from them, and the universal opinion is that Berlin excels by far in Pathology, General Surgery, and General Medicine. Vienna is said to possess advantages over all, in teaching the eye and the ear, with their various diseases. Yet, Berlin boasts of some celebrated men in these branches, of whom I will make mention in a few minutes. Paris is only spoken of as a thing of the past, and as a proof of her present depressed condition, in an educational point of view, is the fact of quite a number of her most distinguished Teachers having left for other countries, in which to practice and teach their profession.

Prof. Von Langerbeck's reputation is world-wide, and it needs but a simple mention of his name, in connection with Surgery, to attract attention and interest. Although approaching seventy years of age, he applies himself as assiduously and continuously as if quite young and strong, to bring himself before the professional world. He commenced his surgical career by performing operations, and that too, successfully, at which other men, less bold, shrugged their shoulders and refused to undertake. Success in such things is well calculated to win reputation, and it seems that the propensity has clung to the Professor in after-life, for daily operations are made which, to a "looker-on," appear dangernosly useless, but it seems as if he had counted the cost, for the results are remarkably successful. Plastic operations are his special delight, and even to-day, I have seen him make two new mouths and a nose, which gave their possessors quite an improved appearance, and will, no doubt, satisfy every want of the deformity. As most of the other Professors, he speaks tolerable English, and in private, is one of the most agreeable of men.

Pathology, as taught by "Prof. Virchow," can certainly not be surpassed in any land, and the interest taken in this very necessary study by almost every American here, is a very noteworthy fact, since amongst our medical men and schools at home, this branch of medicine is comparatively neglected, or if taught at all, very imperfectly so. This careful and almcst universal attention to pathological changes, given by our countrymen, indicates a determination on their part, to improve the present favorable opportunity of acquiring a knowledge of so important a science, hitherto so sadly overlooked in their own country. Being a slow and distinct speaker, and so thoroughly animated by his subject, and so much interested in the progress of his students, "Prof. V." is the especial favorite of all, and especially so with Americans—not only from the admirable manner in which he teaches his subjects, but also, from his great affability and the clear and perfectly articulated speech by which they can, even with but a slight knowledge of the language, catch the idea, if not the full meaning of each word. In this man is combined statesmanship and science—since he occupies, at the same time, one of the first positions in the "German Reichstag," or Congress, and the very first in the science he teaches. Being only fifty-five years of age, he has written more, during this time, upon scientific subjects than any other man of this country. The latest edition of his work on "Cellular Pathology," is in the hands of almost every European physician, and sellers of medical books tell me the demand is still so great that it can only be supplied at an exorbitant rate, when compared with the price of other German literature.

"Von Græfe," whose name occupies the most conspicuous stand in every publication, dating back for ten or fifteen years, upon the Eye and its diseases, and whose reputation made Berlin the world's centre for such studies, and with whom no other man in Germany dared to cope, is now dead, and since then numerous other lesser luminaries have sprung into existence. His successor, "Professor Schweigger," a former pupil, was, shortly after the death of his illustrious predecessor and preceptor, called to the University of Berlin from Heidelberg, where he was occupying a similar chair, to the one now under his control. Although a young man he stands first after Græfe, and bids fair to equal him in all respects. We judge of men, always, by the success of their undertakings, and this rule has brought "Professor S." to follow close upon the reputation of the previous distinguished occupant of the chair of "Diseases of the Eye," in the above-mentioned school. The cases we see daily, in his clinic, are not only such as we meet in every-day practice, but also, those upon whom the more important operations are performed. His treatment of Acute and Chronic Conjunctivitis, with granulated lids, is so simple and so invariably successful, that I propose, at another time, giving it to your readers, for the benefit of their many patients who suffer so terribly from this disease, and whose treatment, especially of the latter, is usually so unsatisfactory.

An examination of the general arrangement of the Eye Wards, and a view of the almost constant attention of assistants and nurses after operations of greater and less importance, impresses upon one, more fully, what he already knows, that, through these means, a successful termination is reached, in as great a degree, as through the skill of the Surgeon himself. A master-head and hand must have superintended the construction of this portion of the hospital.

Berlin also, prides herself upon her specialists, celebrated for their teachings upon "the ear and its diseases," and who have greatly assisted in bringing these branchs of medicine into a separate and distinct and important study. Profs. Lucæ and Weber stand foremost. The latter has recently produced considerable favorable comment, from the profession, through the different journals, from his new mode of treatment of certain affections of the ear, by the division of the "tensor tympani muscle" upon the same principle that certain troubles in the eye, are cured by iridectomy, does this cutting of the tympani muscle, relieve these peculiar complaints of the ear. "Prof. W." remarked to me, that through this operation, he had relieved almost instantly, patients who had suffered from a ten year's "singing in the ear," and as almost constant pain. This "singing," as it is called, is frequently so annoying that the sleep of persons thus effected, is constantly disturbed and unrefreshing, and any mode of treatment, that brings ease and quiet to the sufferer, is certainly a blessing. Most interesting clinics are held daily, where one sees every species of ear diseases, and their various treatment, and can himself make personal examinations, and watch the effect of remedies and operations from day to day.

Prof. Liebreich, "of chloral hydrate" renown, gives a course upon "chemistry and chemical medicine," and in looking over his class, it is easy to percieve, that a large, if not the larger portion, are Americans. He is now devoting himself to the discovery of an anæsthetic, which will completely supercede chloroform, without any of its dangers. Such, at some future time, will be brought into use, and the fortunate discoverer, will receive the praises and thanks, both of his professional colleagues and the world at large.

General medicine in Berlin, is said to surpass the balance of Europe—in fact, a Professor remarked, in my hearing a few days ago, that the manner of teaching, and the clinics of Prof. Frierichs and Traube, are better than anything of the kind, in existence. I have long since found out, that their ideas upon any subject pertaining to the branches which they teach, are held as absolute authority in Germany.

Indeed, lectures upon every specialty, are here given, and numerously attended, and material for their proper study is in the greatest abundance.

To any one anticipating a visit to any portion of Germany, for the study of medicine, I would say, the more German you can read, the better for you—and better still, the more you can speak and read. Numbers of Americans come here, expecting to learn the language after arriving. This is good enough, if you have sufficient time to spend for that purpose—but a great mistake, if you intend entering immediately upon the study for which you come, and have but a limited time in which to do so. Without any knowledge of the language, the first three or four months are completely thrown away. You not only do not understand what is being said, but are in a state of continual mental confusion, by getting wrong ideas.

For those expecting to dissect, it is well to bring an English Anatomy with them, as they will be much better able to proceed with satisfaction—and since, too, the anatomical terms used here in lectures, are exactly the same as with us, with the exception of a different pronunciation. A medical dictionary is also essential. Other English works needed—are but few, as the time is fully occupied otherwise, than by reading to any great extent. Wishing you much success in your journal,

I am yours, most truly,

A. W. Calhoun.

